# Outcomes after traffic injury: mental health comorbidity and relationship with pain interference

**DOI:** 10.1186/s12888-020-02601-4

**Published:** 2020-04-28

**Authors:** I. Pozzato, A. Craig, B. Gopinath, A. Kifley, Y. Tran, J. Jagnoor, I. D. Cameron

**Affiliations:** 1 John Walsh Centre for Rehabilitation Research, Northern Clinical School, Kolling Institute of Medical Research, Faculty of Medicine and Health, The University of Sydney, Corner Reserve Road & Westbourne Street, Royal North Shore Hospital, St Leonards, NSW 2065 Australia; 2 Centre of Healthcare Resilience and Implementation Science, Australian Institute of Health Innovation, Macquarie University, North Ryde, NSW 2109 Australia; 3 The George Institute for Global Health, The University of NSW, Sydney, Australia

**Keywords:** Injury, Depression, Post-traumatic stress, Pain catastrophizing, Pain interference

## Abstract

**Background:**

Mental health symptoms, like depressive mood (DM) and post-traumatic stress (PTS), and pain interference (PI) with daily functioning often co-occur following traffic injury and their comorbidity can complicate recovery. This study aimed to map the course and overlapping trajectories of mental health symptoms, and associations with PI in a traffic injury population.

**Methods:**

In total, 2019 adults sustaining minor-to-moderate traffic injury were recruited within 28 days post-injury and assessed using phone interviews at 1, 6 and 12-months post-injury. Trajectories of DM, PTS and PI were established and relationships between DM, PTS and PI trajectories were explored using dual trajectory modelling. Bio-psychosocial predictors (e.g. pre-injury health, catastrophizing, acute distress, quality of life, social support) of mental health trajectories were investigated.

**Results:**

Up to five typical post-trauma trajectories were identified for DM, PTS and PI. Most people were in a resilient mental health trajectory (over 60%, DM or PTS), or in a chronic PI trajectory (almost 60%) 12 months post-injury. While recovery/resilient mental health trajectories were strongly interrelated (73.4% joint probability and > 94% conditional probabilities), DM/PTS comorbidity in chronic trajectories was not straightforward, suggesting a possibly asymmetric relationship. That is, persistent DM was more likely associated with persistent PTS (90.4%), than vice versa (31.9%), with a 22.5% probability that persistent PTS was associated with none or milder depression (i.e. following a recovery/resilient DM trajectory). An asymmetrical relationship was also found between mental health and PI. The majority of those with persistent PI were likely to be in a recovery/resilient DM/PTS trajectory (almost 70%), but those in a non-resilient DM/PTS trajectory showed a high risk of persistent PI. Predictors of non-resilient mental health trajectories included poorer pre-injury health and social support, and shared factors like acute psychological distress and pain catastrophizing.

**Conclusions:**

Strong interrelations were confirmed between mental health symptoms and PI following traffic injury. However, persistent DM was more strongly linked to persistent PTS, than vice versa. Persistent PI was only linked with persistent DM/PTS in vulnerable subgroups. Early psychiatric/psychological interventions should target elevated psychological distress and negative appraisals in vulnerable individuals, to reduce long-term mental health morbidity/comorbidity and PI.

**Trial registration:**

ACTRN12613000889752.

## Background

Traffic injuries have a very considerable global impact, affecting up to 50 million people every year [1]. Just like other injuries [2], irrespective of their initial severity [3], traffic injuries can result in significant mental health problems [4–6], chronic pain and related interference on daily functioning [7, 8], which often co-occur [9]. Comorbid conditions pose a challenge for injured persons, as well as treating clinicians, because standard unimodal approaches frequently lead to suboptimal outcomes [10]. Past studies of comorbidity have often focused on a subset of problems in specific injury groups (e.g. post-traumatic stress and pain in whiplash) and few studies have explored comorbidity in the transition from acute injury to chronicity [9]. By adopting a person-centred approach [11], further analyses must consider these problems as an integrated system in the person’s recovery course. A thorough understanding of the transition of each condition and their associations over time is crucial to develop evidence-based, tailored, integrated interventions that may lead to improved recovery [12].

Depressive mood (DM) and post-traumatic stress (PTS) are very common mental health symptoms following an accident or injury [5, 13–15], however, most studies have investigated PTS rather than depression. If these DM and PTS symptoms remain elevated, there is an increased risks of disability [16–18] and progression to serious mental disorders (as per Diagnostic and Statistical Manual (DSM) of Mental Disorders criteria), like major depressive disorder (MDD), post-traumatic stress disorder (PTSD), panic disorder and generalized anxiety disorder [5, 19]. Post-injury rates of mental disorder have been reported in the range of 30–50% [5, 14, 20, 21].

Individual course and relationships over time between multiple problems are best captured by person-centred analysis, such as growth mixture models [11, 22]. A few studies have now examined trajectories of mental health following injury [18, 23–27]. Bryant et al., (2015) used latent class analysis (*n* = 1084) to establish trajectories of PTSD severity over a period of 6 years post-injury. They found five trajectories: (a) 4% with chronically high levels (chronic), (b) 6% whose severity levels were reducing (recovery), (c) 8% who were showing increasing PTSD severity but improving at 6 years (worsening/ recovery), (d) 10% who became more severe (worsening) and (e) 73% who had stable low PTSD symptoms across time (resilient) [26]. These trajectories are typical of those found following a trauma of some kind, that is, at least four trajectories (resilient, recovery, chronic and delayed onset), according to a systematic review of 54 studies [25]. Only two studies have investigated mental health trajectories entirely in people with traffic injuries, both focusing on PTS trajectories [23, 24], but other evidence suggests people suffering traffic injuries may differ in their adjustment compared to other traumas [28], with frequent changes in psychiatric diagnosis over time [5]. More studies are warranted to study mental health adjustment following a traffic injury in a comprehensive manner.

Mental health comorbidity is typical after an injury [5, 13, 19, 20], most frequently involving PTSD and MDD [21, 29–31], but the nature of this association remains unclear. Some researchers have explored shared predictors and vulnerability in an effort to clarify whether these conditions are unique or separate constructs [29, 30]. These and other studies [32] have pointed to the importance of bio-psychosocial factors like mental health history, associated physical problems, cognitive bias/perceptions (catastrophic thinking, perceived threat to life), levels of acute distress, the presence of social support and compensation involvement. These above factors have been found to contribute more than demographic and injury factors, in predicting mental health wellbeing after a traffic injury [32]. Other researchers have studied directionality of association over time by looking at reciprocal or directional changes to understand mental health comorbidity [31]. Following traffic injuries, those affected by multiple disorders (e.g. PTSD and MDD) or suffering PTSD showed an increased risk of chronicity in any disorder [5, 29]. Therefore, additional understanding concerning mental health comorbidity patterns, including overlapping trajectories and the existence of modifiable shared risk factors, is needed in this population.

Pain frequently co-occurs with mental health problems [33–35], especially after injury [2, 7, 36], resulting in more complex management of psychiatric care [33, 34, 36] and higher costs [37]. Shared neurobiological underpinning [38] and reciprocal relationships have been found between these conditions, that is, mental health problems increase risk of persistent pain [2, 24, 34, 36] and vice versa [34, 39]. Analysis of mental health-pain relationships among traffic crash survivors is mostly restricted to whiplash cohorts and PTSD [9, 24], despite substantial evidence showing pain influences mental health outcomes [40] and negatively interferes with injury recovery [8].

The impact of pain on the individual’s physical and psychological functioning is known as pain interference [41]. The literature is robust with respect to the need of considering not just pain intensity (personal experience of pain as a symptom) but also pain interference (person’s perception of the extent to which pain limits daily activities) among trauma patients [42]. Pain interference is strongly associated with both pain intensity and psychological symptoms, such as mood problems [43–45]. However, studies of comorbid patterns between pain interference and mental health symptoms following an injury are scarce.

Traffic injuries have a huge global impact and result in substantial (co) morbidity [1], but the limited focus of past research on specific injuries like whiplash and mental disorders like PTSD we believe, has resulted in a deficit in knowledge about how to improve recovery outcomes. There exists a critical need for large prospective research to examine comorbidity of multiple mental health symptoms and associations with pain related trajectories in traffic injury populations, especially minor-to-moderate injuries [3], given troubling prevalence of mental health symptoms following these injuries and related worldwide health burden [46]. Therefore, the current study aimed to investigate: (i) trajectories of DM and PTS and any PI over a 12 month-period post-injury; (ii) the association between DM and PTS; (iii) the association of DM/PTS with PI; and (iv) bio-psychosocial predictors of membership in a mental health trajectory.

It was hypothesized that: (1) at least four trajectories in DM, PTS and PI will be found over a period of 12 months post traffic injury; (2) the probability will be high that participants in a resilient/recovery or chronic trajectory in DM will be respectively a member of resilient/recovery or chronic trajectory in PTS, and vice versa (i.e. mental health trajectories are symmetric and highly interconnected over time), (3) the probability will be high that persistent PI trajectories are strongly associated with chronic/worsening mental health trajectories, and (4) bio-psychosocial variables (e.g. prior mental health issues, acute distress, pain intensity and catastrophizing, social support) will be independent predictors of membership in DM and PTS trajectories, with psychological factors being shared predictors.

## Methods

### Design and procedure

The study employed a multi-site inception cohort design, defined as recruitment within 28 days post-injury from emergency departments, general practitioners, physiotherapy clinics and the claim database of a government insurance regulator in New South Wales (NSW), Australia. Recruitment was ongoing between August 2013 and December 2016. Potential participants were screened, then invited to participate by letter invitation, with the option of opting-out of the study via telephone or email within 1-week of the letter mail out. Verbal consent was obtained over the phone from those who did not opt out. Phone interviews occurred at 1 (i.e. within 4 weeks of injury), 6 and 12-months post-injury. Data were entered on a secure online platform, called Research electronic data capture (REDCap) [47].

Study inclusion criteria include: (i) being 17 years old or more; (ii) sustaining a traffic-related physical injury and being recruited within 28 days of injury; (iii) being a resident of New South Wales (NSW), Australia; (iv) having sufficient English proficiency. People were excluded if they: (i) have sustained a major trauma or catastrophic injury (e.g. spinal cord injury, moderate/severe traumatic brain injury, extensive burn, major amputation); (ii) have sustained a very minor soft tissue injury only (e.g. bruise, abrasion, cut); (iii) have sustained injury due to intentional self-harm; (iv) have experienced the loss of a family member in the crash; (v) have prior cognitive issues impacting on the ability to consent. For details on recruitment, design and procedure see Jagnoor et al. [48].

### Participants

People aged at least 17 years old were invited to participate after sustaining minor-to-moderate traffic-related injury (i.e. musculoskeletal injury, mild traumatic brain injury) over the 42-month period. A total of 2019 participants met inclusion/exclusion criteria (see Additional file 1). Response rates for the 6 and 12-month follow-up were 73.5 and 59.5% respectively. Those with follow-up at 6 or 12-months were older (*p* < 0.0001) and more likely to be born in Australia (p < 0.0001), have tertiary education (p < 0.0001) and report small or no perceived danger of death caused by the accident (p < 0.0001). They did not differ significantly in paid work status but were less likely to be unemployed and more likely to be a student or retired (*p* = 0.0004). They did not differ significantly in sex distribution, comorbidities, self-rated pre-injury health, hospitalization or number of injuries.

### Measures

#### Bio-psychosocial predictors

All predictors of DM/PTS trajectories were collected at the baseline phone interview. The list of potential predictors was guided by prior research on psychological impacts of traffic injury [6, 32]. The final model includes the following factors: (i) Employment at the time of injury which was dichotomized (yes/no). (ii) Self-reported pre-injury health-related quality of life (HRQoL), using the European Quality of Life-5 Dimensions (EQ-5D-3 L) scale that includes five dimensions: mobility, self-care, usual activities, pain/discomfort and anxiety/depression [49]. Each domain is measured by a 3-point Likert scale (0-no problem, 1-some problems, 3-extreme problems). An overall summary score, on a range between − 1 and 1 was calculated, based on EQ-5D-3 L value sets for perceived value of the possible combinations of health states reported. (iii) Post-injury HRQoL was measured using the Short Form 12 (SF-12), a valid and reliable self-reported health survey [50], providing a distinct physical and mental component summary scores. (iv) Pain intensity was measured using an 11-point Likert scale (0-no pain to 10-worst pain ever) [51]. The following psychosocial predictors were also included: (v) Perceived danger of death during the road crash was assessed on a 5-point Likert scale (0-none to 5-overwhelming). (vi) Satisfaction with social relationships was assessed using a 5-point Likert scale (1-poor to 5-excellent). (vii) Pain-related psychological distress (e.g. feeling helpless about their pain) was measured using the Pain Catastrophizing Scale (PCS) [52]. The PCS is a 13-item 5-point Likert scale (0–4), a range of 0–52 with scores 34 or above indicating severely elevated pain-related catastrophic thinking. However, due to a transposing error, a 6-point Likert scale (0-not at all to 5-all the time) was used rather than the usual 5-point scale, resulting in totals ranging between 0 and 65. These totals were rescaled so that the final score would lie on the published range of 0–52. This minor alteration of the Likert scoring did not alter the outcome of the analyses [6]. The PCS has been shown to have adequate reliability, validity and internal consistency [52].

Table 1 shows baseline socio-demographic (e.g. age, sex, education) and injury-related factors (e.g. time in hospital, type and number of injuries, road user type). Based on previous evidence [32] and supplementary analysis showing inclusion of these factors didn’t alter study findings, these were not included in the final predictive model (also given they are not modifiable). Although the study collected information on injury compensation, further exploration of the impact of this factor was not included in this paper.

**Table 1:** Baseline socio-demographic, crash and injury-related characteristics of study participants (*N* = 2019)

Variables	Mean (SD), range or N (%)
**Age** in years	41.1 (16.5), 17–92
**Sex**	
Male	1305 (64.6)
Female	714 (35.4)
**Country of birth**	
Australia	1434 (71.0)
United Kingdom	127 (6.3)
New Zealand	58 (2.9)
Other	400 (19.8)
**Highest educational level**	
University or tertiary education	789 (39.1)
Technical or other further education	488 (24.2)
Secondary	614 (30.4)
Primary or pre-primary	126 (6.3)
**Paid work or self-employment at time of injury**	
Yes	1533 (75.9)
No	486 (24.1)
**Pre-injury EQ. 5D summary score**	0.93 (0.14) -0.18-1
**Pre-injury co-morbidities**	
Yes	1140 (56.5)
No	878 (43.5)
**Pre-injury health rating**	
Excellent	779 (38.6)
Very good	740 (36.7)
Good	372 (18.4)
Fair	111 (5.5)
Poor	17 (0.8)
**Road user role in crash**	
Driver	723 (35.9)
Passenger	204 (10.1)
Motorbike driver	622 (30.8)
Pillion passenger	6 (0.3)
Bicyclist	299 (14.8)
Pedestrian	139 (6.9)
Skateboarder	24 (1.2)
**Areas with injuries**	
Head or face	603 (29.9)
Neck	667 (33.0)
Spine or back	777 (38.5)
Torso	901 (44.6)
Upper extremity	1367 (67.7)
Lower extremity	1157 (57.3)
**Perceived danger of death in crash**	
Overwhelming	207 (10.5)
Great	313 (15.8)
Moderate	391 (19.8)
Small	389 (19.7)
None	680 (34.3)
**Self-reported time spent in hospital**	
Did not attend	58 (2.9)
< 12 h	935 (46.3)
> 12 h – 24 h	311 (15.4)
2–6 days	507 (25.1)
7 days or more	207 (10.3)
**Any pain since injury**	
Yes	1755 (86.9)
No	264 (13.1)
**Baseline average pain intensity rating** (in those with pain)	4.9 (2.3) 0–10

Note: These demographic/ injury data have been presented in prior publications

#### Post-traumatic stress (PTS) symptoms

The Impact of Events Scale Revised (IES-R) was used to measure the presence of PTS symptoms at 1, 6 and 12-months post-injury via phone interviews. The IES-R is a 22-item self-report measure with acceptable reliability and validity [53] that has been validated in people with traffic injuries [54]. Participants were asked to indicate their degree of PTS during the past 7 days related to their recent road crash on a 5-point scale (0-not at all to 4-extremely) for subscales avoidance, intrusion and hyperarousal. Domains are scored (range 0–4) by determining the mean item score. The maximum mean score on each of the three subscales is ‘4’, therefore the maximum ‘total mean’ IES-R score is '12' (the sum of the 3 subscales). Higher scores indicate higher levels of PTS. Based on available norms, a total mean score of ≥4.5 (i.e. ≥33 of the total score of 88) represents clinically elevated PTS, that is, a probable PTSD diagnosis [21, 53].

#### Depressive mood (DM) symptoms

The 7-item depression subscale of the Depression Anxiety Stress Scale-21 (DASS-21) was used to assess the presence of DM symptoms at 1, 6 and 12-months post-injury via phone interviews. DASS-21 is a 21-item scale providing an overall assessment of psychological distress based on DSM criteria [55], including three subscales of 7-item each: depressive mood, anxiety and perceptions of stress [56]. The depression subscale includes seven 4-point Likert items (0–3) assessing DM symptoms over the past week: dysphoria, hopelessness, devaluation of life, self-deprecation, lack of interest, anhedonia and inertia. Depression scores are calculated by summing all 7 items and range from 0 to 21. The DASS-21 has acceptable reliability and validity [56]. Based on available norms for DASS-21 depression subscale in people following traffic injuries, scores of ≥5 out of 21 (i.e. ≥10 out of 42 if scores are multiplied by 2 according to the original DASS-42) represent clinically elevated DM, that is, a probable MDD diagnosis [21].

#### Pain interference (PI)

PI was assessed at 1, 6 and 12-months post-injury using item 8 from the SF-12 health survey [50] administered over the phone, which is a 5-point Likert scale (0-not at all to 5- extremely) (“During the past 4 weeks, how much did pain interfere with your normal work including work outside the home and housework”). We dichotomized PI as any versus no pain interference for the GMM analyses because there were only five levels of response.

### Statistical analysis

Growth mixture modelling (GMM) and dual trajectory analyses are person-centred approaches [22] that were employed to achieve study objectives. Growth mixture modelling (GMM) in Mplus version 7.3 [57] was used to determine trajectories for change over time in DM, PTS and PI, adjusting for significant predictors of trajectory assignment (conditional model). Unconditional models without covariates were examined initially, however the trajectory assignments improved after including significant predictors in the modelling. Growth parameters were allowed to vary, with constraints applied if required due to computation issues. Potential effects of predictors on growth parameters were examined initially but were not substantially affecting trajectory assignments. GMM was estimated under missing data theory using all available data, thus allowing for missing follow-up on the outcome measures.

The optimal number of trajectories was determined from information criterion indices, such as Akaike information criterion (AIC), Bayesian information criterion (BIC) and entropy values [58]. Methods recommended for determining the number of trajectories in GMM analyses include the model that best meets four criteria: (i) the smallest indices for the Bayesian Information Criterion (BIC) and the Akaike information criterion (AIC); (ii) higher entropy values; (iii) significant Vuong-Lo-Mendell-Rubin (VLMR), Lo–Mendell–Rubin (LMR) and parametric bootstrap (PB) likelihood ratio test (LRT) statistics, and (iv) interpretability of the model [58].

Estimated trajectory assignments were used to calculate joint and conditional probabilities of membership in DM trajectories given PTS or PI, and vice versa. Findings were confirmed using dual trajectory modelling analyses that calculated the relationships and concurrence between DM, PTS and PI trajectories [22]. The output from the dual trajectory analyses was the probability of linked memberships in the DM, PTS and PI trajectories. Data were analysed using Mplus version 7.3 software (https://www.statmodel.com/), Statistical Analysis System (SAS) version 9.4 and R version 3.5.1.

## Results

Compared with those without 12-month follow up, those with follow up had lower baseline DM symptoms (mean DASS depression scores: 3.48 (3.20–3.77) vs 4.37 (3.99–4.75)) and PTS symptoms (mean IESR scores: 3.35 (3.18–3.52) vs 4.06 (3.82–4.29)), but similar baseline levels of PI (mean PI scores: 2.05 (1.97–2.13) vs 2.09 (1.99–2.19)). While rates of DM and PTS decreased over time, at 12 months around 20% still had clinically elevated DM and around 17.5% clinically elevated PTS (Table 2). Overall, at 12-month post-injury, one in four (24.8%) people were suffering persistent DM or PTS and over 40% reporting any PI.

**Table 2:** Proportions of individuals with elevated symptoms of depressive mood (DM) and post-traumatic stress (PTS), and proportions with any perceived pain interference (PI) at baseline, 6 months and 12 months

	Elevated DM (DASS depression score > = 5/21 or > = 10/42)	Elevated PTS (IESR total meanscore > = 4.5)	Elevated DM and/or PTS	Any PI
	*N (%)*	*N (%)*	*N (%)*	*N (%)*
Baseline (*n* = 2019)	590 (29.3)	678 (33.9)	833/ 2006 (41.5%)	1667 (82.7)
6 months (*n* = 1484)	362 (24.4)	309 (20.9)	453/ 1477 (30.7%)	735 (49.5)
12 months (*n* = 1201)	240 (20.2)	207 (17.5)	293/ 1183 (24.8%)	502 (41.8)

### Trajectories of DM, PTS and PI

**Fig. 1:**
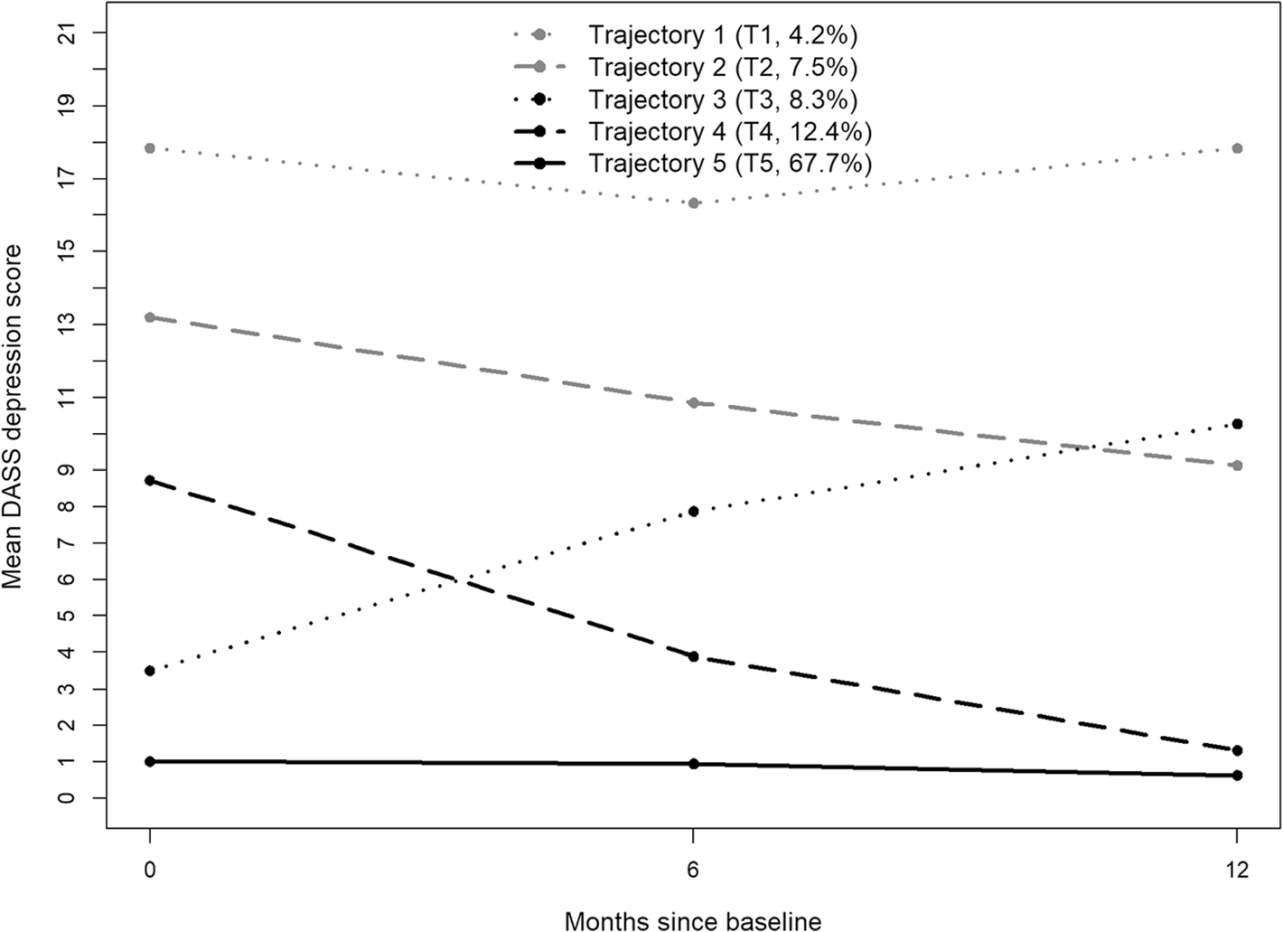
Trajectories of Depressive Mood (DM), based on the Depression Anxiety Stress Scale (DASS) depression subscale. Trajectory 1 (T1): Chronic; Trajectory 2 (T2): Moderate-Chronic; Trajectory 3 (T3): Worsening; Trajectory 4 (T4): Recovery; Trajectory 5 (T5): Resilient

Based on information criterion indices of GMM trajectories (see Additional file 2), a five-trajectory solution was considered the best fit for DM, a four-trajectory solution for PTS and a three-trajectory solution for PI. Figure 1 shows five DM trajectories. There was a probability of 67.7% of belonging to a trajectory with stable low DM (T5, resilient; most likely *n* = 1335), 12.4% probability of belonging to a trajectory with improving DM (T4, recovery; most likely *n* = 244), 8.3% probability of belonging to a trajectory with worsening DM (T3, worsening; most likely *n* = 164), 7.5% in a trajectory with moderate-chronic DM (T2, moderate-chronic, most likely *n* = 147), and 4.2% in a high DM trajectory (T1, chronic; most likely *n* = 83).

**Fig. 2:**
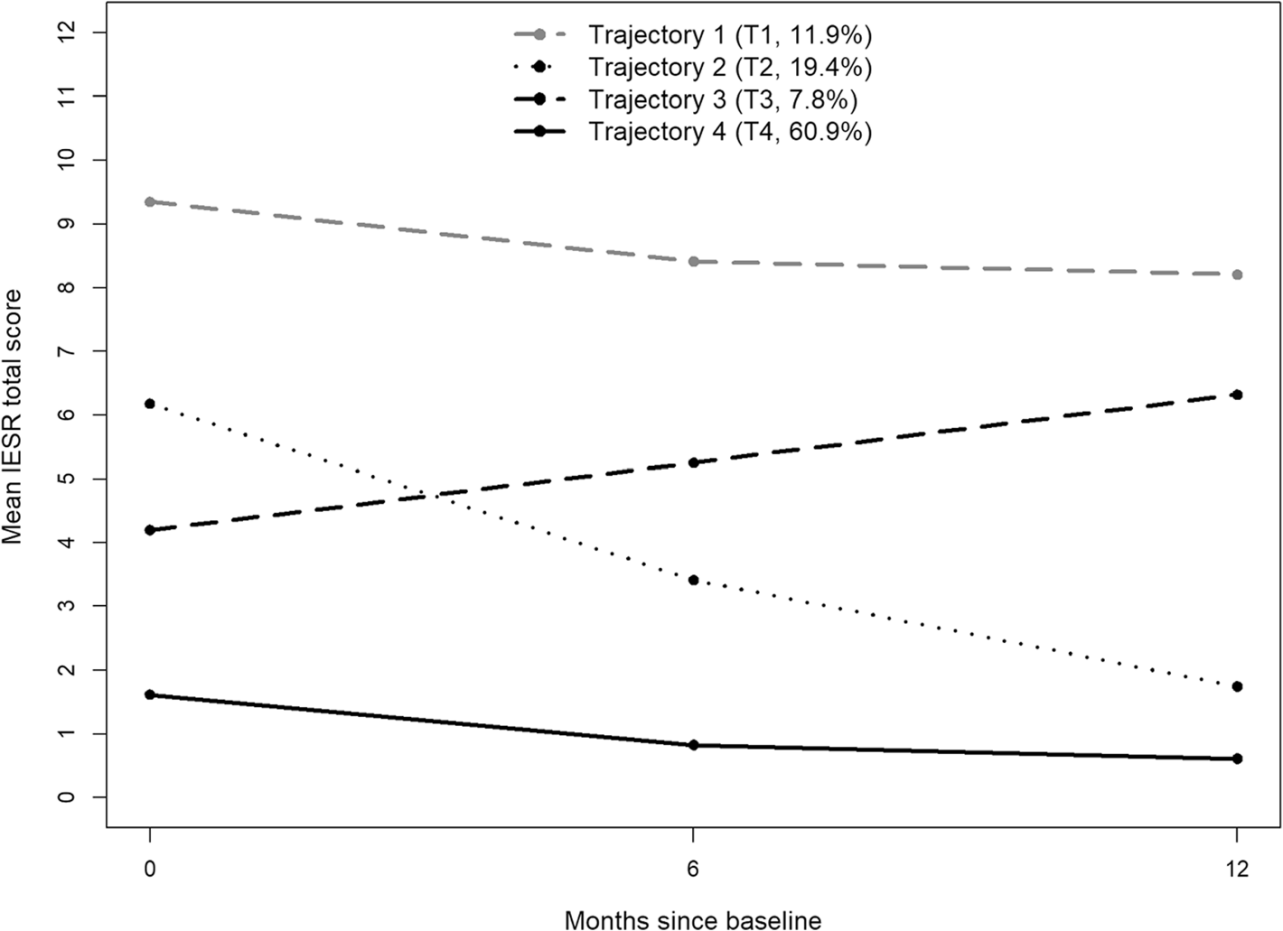
Trajectories of Post-traumatic stress (PTS), based on the Impact of Events Scale Revised (IES-R). Trajectory 1 (T1): Chronic; Trajectory 2 (T2): Recovery; Trajectory 3 (T3): Worsening; Trajectory 4 (T4): Resilient

**Fig. 3:**
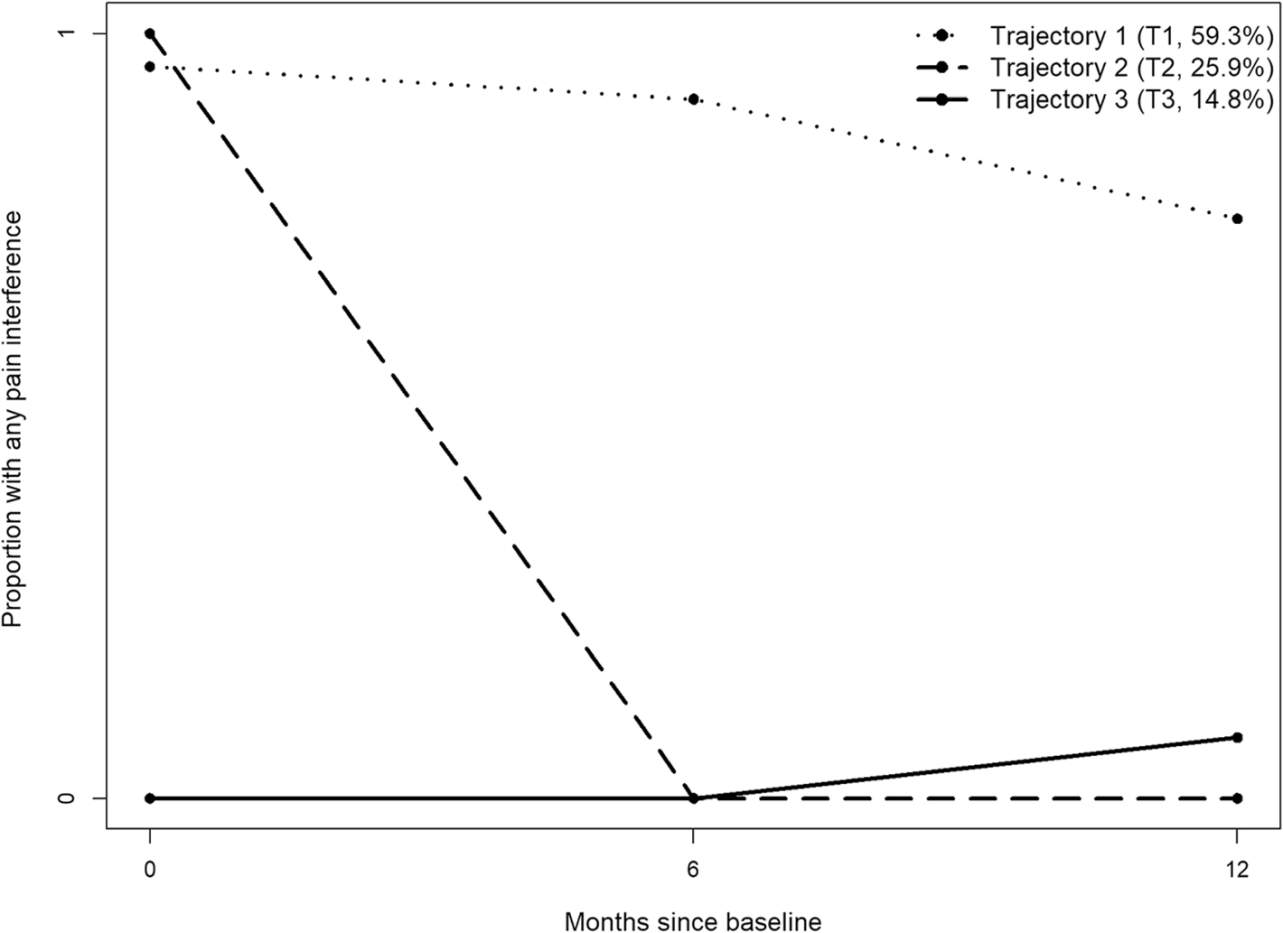
Trajectories of Pain interference (PI), based on the presence of any pain interference (SF-12). Trajectory 1 (T1): Chronic; Trajectory 2 (T2): Recovery; Trajectory 3 (T3): Resilient

Figure 2 shows four PTS trajectories, with a probability of 60.9% of belonging to a trajectory with stable low PTS (T4, resilient; most likely *n* = 1206), a 7.8% probability of belonging to a trajectory with increasing PTS (T3, worsening; most likely *n* = 154), a probability of 19.4% of belonging to a trajectory with reducing PTS over time (T2, recovery; most likely *n* = 384), and a 11.9% probability of belonging to a trajectory with stable high levels of PTS over time (T1, chronic; most likely *n* = 236). By contrast, only three trajectories were found for PI (Fig. 3) with a probability of 59.3% of belonging to a trajectory with any persistent PI (T1, chronic; likely *n* = 1198); a probability of 25.9% of belonging to a trajectory with reducing PI over time (T2, recovery; likely *n* = 523), and a 14.8% probability of belonging to a trajectory with stable low levels or no PI (T3, resilient; likely *n* = 298).

### Predictors of mental health trajectory

With the DM resilience trajectory 5 as the reference (see Additional file 3), factors found to be predictive of membership in the DM trajectories (T1–4) included poorer pre-injury health (all trajectories *p* < 0.001), elevated PTS at baseline (all trajectories, *p* < 0.001), pain catastrophizing (all trajectories, at least *p* < 0.05), dissatisfaction with social life (all trajectories, at least *p* < 0.05), poorer physical health related-quality of life (T3, worsening, *p* < 0.001) and poorer mental health related-quality of life (all trajectories, *p* < 0.001). With the PTS resilience trajectory 4 as the reference, factors found to be predictive of membership in the PTS trajectories (T1–3) included elevated DM at baseline (all trajectories, *p* < 0.001), high pain intensity (all trajectories except T2, recovery, at least *p* < 0.05), pain catastrophizing (all trajectories, at least *p* < 0.001) and poorer mental health related-quality of life (all trajectories, *p* < 0.001).

### Relationships between DM and PTS

Table 3 shows the joint and conditional probabilities between DM, PTS and PI. It was anticipated that a high probability of being in a recovery/resilient trajectory or a chronic trajectory would occur for both DM and PTS. The largest joint probability for DM and PTS consisted of people who were members of the recovery/resilient (T2/T4) PTS and the recovery/resilient (T4/T5) DM trajectories (56.2 + 7.8 + 7.0 + 2.4 = 73.4% of the sample). Being a member of a chronic/worsening trajectory in DM and PTS would be also expected, but this was not the case. Instead, there was only a 13.1% probability of belonging to a DM chronic (T1), moderate chronic (T2) or worsening (T3) trajectory and being a member of a PTS chronic (T1) or worsening (T3) trajectory, and a similar joint probability of 13.4% of belonging to a chronic/worsening trajectory and being a member of a recovery/resilient trajectory for either DM or PTS.

**Table 3:** Joint and conditional probabilities between depressive mood (DM) and post-traumatic stress (PTS) trajectories, and between depressive mood (DM) trajectories and pain interference (PI) trajectories

	DM trajectories				
	Chronic (Trajectory 1)	Moderate-Chronic (Trajectory 2)	Worsening (Trajectory 3)	Recovery (Trajectory 4)	Resilient (Trajectory 5)
Joint probability of DM and PTS					
**PTS trajectories**					
Chronic (Trajectory 1)	3.8	4.4	1.1	2.2	0.5
Worsening (Trajectory 3)	0.2	0.9	2.7	0.7	3.2
Recovery (Trajectory 2)	0.1	2.0	2.4	7.0	7.8
Resilient (Trajectory 4)	0.0	0.2	2.1	2.4	56.2
Conditional probability of DM given PTS/ of PTS given DM (**in bold**)					
**PTS trajectories**					
Chronic (Trajectory 1)	31.9/ **90.4**	36.6/ **58.5**	8.9/ **12.9**	18.7/ **18.0**	3.8/ **0.7**
Worsening (Trajectory 3)	3.2/ **6.0**	11.7/ **12.2**	34.4/ **32.5**	9.1/ **5.7**	41.6/ **4.8**
Recovery (Trajectory 2)	0.8/ **3.6**	10.2/ **26.5**	12.6/ **29.4**	36.2/ **56.6**	40.2/**11.5**
Resilient (Trajectory 4)	0.0/ **0.0**	0.3/ **2.7**	3.4/ **25.1**	4.0/ **19.7**	92.2/ **83.0**
Joint probability of DM and PI					
**PI trajectories**					
Chronic (Trajectory 1)	3.9	6.8	6.9	8.5	32.5
Recovery (Trajectory 2)	0.1	0.3	0.9	3.2	21.6
Resilient (Trajectory 3)	0.2	0.2	0.5	0.7	13.5
Conditional probability of DM given PI/ of PI given DM (**in bold**)					
**PI trajectories**					
Chronic (Trajectory 1)	6.7/ **92.8**	11.7/ **91.8**	11.8/ **83.5**	14.4/ **68.4**	55.4/ **48.1**
Recovery (Trajectory 2)	0.1/ **1.2**	1.4/ **4.8**	3.5/ **11.0**	12.4/ **26.2**	82.6/ **32.0**
Resilient (Trajectory 3)	1.6/ **6.0**	1.7/ **3.4**	3.0/ **5.5**	4.4/ **5.3**	89.3/ **19.9**

Note: Probabilities are expressed in %. Figures in bold pertain to conditional probabilities of PTS given DM and conditional probability of PI given DM, respectively

Joint probabilities are based on modelling estimations so the total probability does not add exactly to 100%

Conditional probabilities are based on modelling estimations. For DM given PTS, the rows add to approximately 100%, while for PTS given DM, columns add to approximately 100%. This is also the case for the conditional probabilities for DM and PI

Probabilities for the association between PTS and PI trajectories are provided as supplementary material given they were similar to the probabilities for DM and PI trajectories

The conditional probabilities for DM and PTS provide some explanation for the above finding. As expected, DM given PTS and PTS given DM conditional probabilities were high for membership in resilient trajectories, 92.2% for DM given PTS and 83% for PTS given DM. While there was a very high conditional probability of 90.4% for membership in a PTS chronic trajectory given DM, there was only a 31.9% probability of membership in the chronic trajectory for DM given PTS. Further, there was a 73.2% conditional probability of membership in a recovery or resilient (T4/T5) DM trajectory if one belonged to a chronic or worsening (T1/T3) PTS trajectory (i.e. a 22.5% probability among those following a chronic PTS trajectory only). This was unexpected.

### Relationships between mental health and PI

It was predicted that persistent PI will be strongly associated with chronic/worsening mental health trajectories. By contrast, the joint probability analysis showed an asymmetrical association, that is, the largest group consisted of people who were members of the chronic (T1) PI and the resilient (T5) DM (32.5% of the sample) or recovery/resilient (T4/T5) DM (41% of the sample). This was also confirmed by the conditional probability analysis of DM given PI showing the majority (69.8%) of participants in the chronic (T1) PI trajectory were in a recovery/resilient (T4/T5) DM trajectory. Similarly, the conditional probability analysis of PI given DM showed that if one was a member of the resilient (T5) DM trajectory, they had a 48.1% probability of being in a chronic PI trajectory (T1). In contrast, for PI given DM, there was a very high probability of being in a chronic PI trajectory (T1) if in a chronic (T1) or moderate-chronic (T2) or worsening (T3) DM trajectory (92.8% or 91.8% or 83.5% respectively). Finally, for DM given PI, there was a very high probability of being in the resilient (T5) DM trajectory if one was in the resilient (T3) or recovery (T2) PI trajectory (89.3% or 82.6% respectively). Analogous asymmetric associations were found between PTS and PI (see Additional file 4).

## Discussion

This study is the first of its kind to use a person-centred analysis to examine comorbidity patterns of prevalent mental health symptoms following minor-to-moderate traffic injuries and exploring relationships between mental health and interference of chronic pain on individual’s functioning over time in a large prospective cohort of road crash survivors.

These results strongly confirm that people sustaining minor-to-moderate traffic injury face high risks of mental distress and pain-related interference with various activities of daily living. After 4 weeks post-injury, over 80% reported their pain interfered with daily functioning, over 30% had clinically elevated PTS symptoms and almost 30% had clinically elevated DM, reflecting a high probability of meeting diagnostic criteria for PTSD and MDD disorders respectively [21]. As expected, these rates reduced with time, however, at 12 months post-injury over 40% continued to report that pain was limiting their daily functioning and many still reported elevated depressive mood and/or post-traumatic stress symptoms (i.e. 17.5% PTS and 20% DM). These findings are in line with 12-month rates of mental disorders (30%) found in the two largest injury studies, each study including over 1000 traffic crash survivors [14, 20].

### Trajectories of DM and PTS, and predictors

Galatzer-Levy et al. (2018) identified, based on the results of 54 independent studies, what they called prototypical resilience trajectories following a trauma: stable low distress or resilience, chronically high distress, worsening distress and reducing distress or recovery [25]. Our trajectory findings confirmed these four prototypical trajectories in the DM and PTS patterns of response to a traffic injury (hypothesis 1 confirmed) and strengthen the conclusion that the majority of individuals follow a resilient mental health trajectory [26, 29].

As found in previous research [29], shared psychological predictors were found for DM and PTS non-resilient trajectories (hypothesis 4 confirmed) that could be readily assessed and addressed in psychiatric/psychological clinical contexts as soon as possible after the injury (i.e. ideally within 1 or 2 months post-injury). The presence of shared predictors supports the assumption of a shared vulnerability [9] and strong interrelations between these conditions following a traffic injury. For instance, catastrophizing styles of thinking is generally related to increased risk of MDD and PTSD [6] and was also found to be a shared predictor of chronic/worsening DM/PTS trajectory membership. The presence of elevated DM and PTS symptoms within 4 weeks of the injury was a strong predictor of poor mental health at 12 months, and should alert clinicians to the need of early intervention [5]. Additionally, dissatisfaction with one’s social life also predicted poor outcomes, reinforcing the importance of social support/engagement as a buffer against poor mental health, especially in people injured and likely to have disability that may become a mobility barrier. Among biological predictors, poorer pre-injury health raises a red flag for risk of elevated DM, while increased pain intensity predicted a high risk of PTS symptoms, underlining the need for integrated pathways of care for injured people with mental health problems if they also have comorbid chronic pain or pre-existing medical conditions [10].

These findings indicate mental health vulnerability and resilience following traffic injury are better interpreted ‘in terms of interactions between biological, emotional, cognitive, behavioural and environmental factors’ [59]. Within this framework, mental distress symptoms and negative thinking associated with a physical injury and pain are shared factors that may help identify less resilient individuals [9, 60, 61].

### Comorbidity patterns between DM and PTS

Previous research on mental health impacts of trauma revealed that PTSD and MDD highly co-occur over time [5, 29–31]. In support, our dual trajectory joint and conditional analyses of the relationships between DM and PTS revealed that recovery/resilient DM trajectories were strongly associated with recovery/resilient PTS trajectories following traffic injury, meaning that resilient individuals are likely to be resilient for both symptoms. Interestingly, relationships between chronic PTS and DM trajectories were more complex. There was a very high conditional probability (90.4%) of having severe PTS symptoms if one had severe depressive mood, though this was not the case for the reverse. There was a much lower probability (31.9%) of having severe depressive mood symptoms if one had severe PTS symptoms. Based on these findings, hypothesis 2 was only partially confirmed.

These results challenge the notion that the risk of developing chronic MDD and PTSD symptoms is symmetrical (i.e. if you have PTSD you will have MDD and vice versa). However, directions of this association cannot be clearly explained by these data. On one hand, our findings of persistent PTS in almost all those with persistent DM could support previous studies that showed that suffering PTSD increases risk for other chronic mental health disorders [5, 30], as well as confirming the existence of comorbid PTSD/depression following traffic injury [29]. But, it could equally support the hypothesis of pre-existing [62] or first-onset elevated MDD after the injury increasing susceptibility for chronic PTSD [31]. On the other hand, these data show that people can follow a favourable trajectory for DM even with persistently elevated PTS (i.e. those with persistent PTS have a 73.2% probability of following a recovery or resilient DM trajectory). This could suggest that after a traffic injury PTS can also occur in isolation or in association with milder forms of depression having a favourable course independently of PTS, as observed by previous findings [29].

All in all, these results, in addition to shared predictive factors supporting shared vulnerability, confirm a robust, possibly asymmetric, association between PTS and DM symptoms following a traffic injury. Possible interpretations of PTS/DM comorbidity may be that a unique distress construct exists following trauma [19, 29], or perhaps that PTS/DM co-occurrence constitutes a distinct trauma-related phenotype, with specific biological correlates and poorer prognosis than a single disorder [63]. Undeniably, comorbidity cases would benefit from early identification and flexible classification systems and treatment options targeting depressive mood and traumatic distress, [22, 63] preferably as early after the injury as possible. Treatment should also be offered at least up to 12 months post-injury, as our findings suggest that a person may, for instance, be recovering in terms of their depressive mood, but be deteriorating in terms of PTS symptoms. The reverse situation seems less likely based on our findings.

### Relationships between mental health and pain interference (PI)

Given the recognised interactive influence between mental health symptoms and pain on recovery from injury [2], it was a concern that there was almost a 60% probability of membership in a trajectory that reported interference in functioning due to pain. Unexpectedly, the dual trajectory modelling findings indicate asymmetrical relationships between pain interference and mental health (both DM and PTS) following a traffic injury (hypothesis 3 was not confirmed). That is, the largest part of the sample (41%) had ongoing PI but good psychological functioning (joint probability). Similarly, almost 70% of those in a chronic PI trajectory were members in the recovery or resilient DM/PTS trajectory and nearly 50% of psychologically resilient survivors had ongoing pain interference (conditional probabilities). These findings demonstrate that people can function well even with the presence of chronic pain, and that other factors may be associated with the persistence of PI following injury other than mental health. In contrast, those in a non-resilient DM/PTS trajectory (i.e. chronic, moderate-chronic and worsening), although being a minority of the sample, were very likely to also follow a chronic PI trajectory, indicating that persisting mental health issues in vulnerable subgroups are associated with increased risk of persistent PI [2, 24, 36]. Instead, those in a resilient PI trajectory were likely to also follow a resilient DM/PTS trajectory. Overall, these findings reveal a high risk of interference of chronic pain with daily functioning for those with chronic mental health symptoms after traffic injury, but not vice versa, reiterating how important it is to address comorbid mental health symptoms early, to reduce long-term risk of prolonged PI in these vulnerable groups.

### Limitations

Although the large sample size, the multisite approach, thus increased patient heterogeneity, the inception cohort design and the use of validated questionnaires are strengths of this study, there are limitations requiring discussion. These include the loss to follow-up, especially at 12 months, the availability of only single items to measures some of the constructs such as pain interference, the presence of unmeasured bias (for instance unmeasured psychological factors), the lack of detailed information on pre-injury mental health morbidity and psychological/psychiatric interventions or any other interventions received during the duration of the study. Also, a standard classification for injury severity (e.g. ISS, AIS, MAIS) was not included in this study, but proxies were used (e.g. time in hospital). Further, while it is accepted that the influence of injury compensation on recovery is an important but complex issue that will influence study findings [61], this was beyond the focus of the present paper, and will be addressed in future analyses. Finally, interrelations between distinct but possibly related symptoms, such as DM and PTS, should be interpreted with caution, as these findings do not offer information on direction, causation or level of dependence of constructs.

Future research should investigate temporal dynamics (e.g. to disentangle contemporaneous change or causality) of the prevalent traffic injury consequences, as well as explore strategies to reduce loss to follow-up, improve the validity of long-term outcomes, improve reproducibility of trajectories and thus generalize results to a wider community of adults with traffic-related injury and other trauma/injury populations. Additional work is also required to clarify any differences in psychological adjustment and comorbidity over time between people with severe [64] and non-severe injuries due to a traffic crash.

## Conclusions

Most injured people (over 60%) showed high psychological resilience following a minor-to-moderate traffic injury, but the same proportion of individuals still suffered persistent interference of pain in daily functioning of any level 12 months post-injury. Chronic mental health problems occurred in vulnerable groups, with persistent depression being more strongly associated with persistent PTS than vice versa. While those in a chronic PI trajectory generally showed adaptive psychological functioning, those following a non-resilient mental health trajectory, although a minority, showed a very high risk of chronic PI. Predictors of non-resilient mental health trajectory membership were bio-psychosocial factors, with shared factors being acute elevated psychological symptoms (i.e. within a month post-injury) and catastrophizing thinking styles. Addressing these factors will assist in the identification of less resilient individuals and are readily modifiable in a psychiatric/clinical psychology context. Overall, by confirming mental health comorbidity and strong asymmetric relationships with PI, our findings strengthen the need for early person-centred interventions and integrated management of these patients to reduce untreated conditions following traffic injury. Interventions are best individually tailored to vulnerable subgroups exhibiting co-occurring problems, targeting early psychological reactions to trauma and cognitive thinking styles (i.e. catastrophizing), and more comprehensively, addressing the bio-psychosocial context that influences resiliency and recovery from adversity.

## Supplementary information

**Additional file 1.** Flowchart of study participation.

**Additional file 2.** Fit Indices for 3–6 trajectory growth mixture models for DASS depressive mood (DM), IES-R post-traumatic stress (PTS), and SF-12 pain interference (PI). Five trajectories were chosen as the best fit for DM, four trajectories for PTS, and three for PI.

**Additional file 3.** Predictors of depressive mood (DM) and post-traumatic stress (PTS) trajectories.

**Additional file 4.** Joint and conditional probabilities* between post-traumatic stress (PTS) trajectories and pain interference (PI) trajectories.

## Data Availability

The datasets used and/or analysed during the current study are available from the corresponding author on reasonable request.

## Abbreviations

DASS-21: Depression Anxiety Stress Scale-21
DSM: Diagnostic and Statistical Manual of Mental Disorders criteria
EQ-5D-3 L: European Quality of Life-5 Dimensions
GMM: Growth mixture modelling
IES-R: Impact of Events Scale Revised
MDD: Major depressive disorder
NSW: New South Wales
PCS: Pain Catastrophizing Scale
PI: Pain interference
PTS: Post-traumatic stress
PTSD: Post-traumatic stress disorder
QOL: quality of life
REDCap: Research electronic data capture
SAS: Statistical Analysis System
SF-12: Short Form 12

## References

[1] World Health Organisation (2018). Global status report on road safety 2018.

[2] Giummarra MJ, Casey SL, Devlin A, Ioannou LJ, Gibson SJ, Georgiou-Karistianis N (2017). Co-occurrence of posttraumatic stress symptoms, pain, and disability 12 months after traumatic injury. Pain Rep.

[3] Gopinath B, Jagnoor J, Elbers N, Cameron ID (2017). Overview of findings from a 2-year study of claimants who had sustained a mild or moderate injury in a road traffic crash: prospective study. BMC Res Notes.

[4] Craig A, Tran Y, Guest R, Gopinath B, Jagnoor J, Bryant RA (2016). Psychological impact of injuries sustained in motor vehicle crashes: systematic review and meta-analysis. BMJ Open.

[5] Kenardy J, Edmed SL, Shourie S, Warren J, Crothers A, Brown EA (2018). Changing patterns in the prevalence of posttraumatic stress disorder, major depressive episode and generalized anxiety disorder over 24 months following a road traffic crash: results from the UQ SuPPORT study. J Affect Disord.

[6] Craig A, Elbers N, Jagnoor J, Gopinath B, Kifley A, Dinh M (2017). The psychological impact of traffic injuries sustained in a road crash by bicyclists: a prospective study. Traffic Inj Prev.

[7] Mayou R, Bryant B (2002). Outcome 3 years after a road traffic accident. Psychol Med.

[8] Gopinath B, Jagnoor J, Kifley A, Nicholas M, Blyth F, Kenardy J (2019). Differential predictors of pain severity over 12 months following noncatastrophic injury sustained in a road traffic crash. J Pain.

[9] McLean SA, Clauw DJ, Abelson JL, Liberzon I (2005). The development of persistent pain and psychological morbidity after motor vehicle collision: integrating the potential role of stress response systems into a biopsychosocial model. Psychosom Med.

[10] Edmond SN, Heapy AA, Kerns RD (2019). Engaging mental health professionals in addressing pain. JAMA Psychiatry..

[11] Howard MC, Hoffman ME (2018). Variable-centered, person-centered, and person-specific approaches: where theory meets the method. Organ Res Methods.

[12] Samoborec S, Ruseckaite R, Ayton D, Evans S (2018). Biopsychosocial factors associated with non-recovery after a minor transport-related injury: a systematic review. PLoS One.

[13] O’Donnell ML, Creamer M, Pattison P, Atkin C (2004). Psychiatric morbidity following injury. Am J Psychiatry.

[14] Bryant RA, O'donnell ML, Creamer M, McFarlane AC, Clark CR, Silove D (2010). The psychiatric sequelae of traumatic injury. Am J Psychiatry.

[15] Papadakaki M, Ferraro OE, Orsi C, Otte D, Tzamalouka G, Von-der-Geest M (2017). Psychological distress and physical disability in patients sustaining severe injuries in road traffic crashes: results from a one-year cohort study from three European countries. Injury..

[16] O'Donnell ML, Varker T, Holmes AC, Ellen S, Wade D, Creamer M (2013). Disability after injury: the cumulative burden of physical and mental health. J Clin Psychiatry.

[17] Kenardy J, Heron-Delaney M, Warren J, Brown EA (2015). Effect of mental health on long-term disability after a road traffic crash: results from the UQ SuPPORT study. Arch Phys Med Rehabil.

[18] Haagsma J, Van Beeck E, Toet H, Polinder S (2013). Posttraumatic stress disorder following injury: trajectories and impact on health-related quality of life. J Depress Anxiety.

[19] Grant DM, Beck JG, Marques L, Palyo SA, Clapp JD (2008). The structure of distress following trauma: posttraumatic stress disorder, major depressive disorder, and generalized anxiety disorder. J Abnorm Psychol.

[20] Mayou R, Bryant B (2001). Outcome in consecutive emergency department attenders following a road traffic accident. Br J Psychiatry.

[21] Guest R, Tran Y, Gopinath B, Cameron ID, Craig A (2018). Prevalence and psychometric screening for the detection of major depressive disorder and post-traumatic stress disorder in adults injured in a motor vehicle crash who are engaged in compensation. BMC Psychol.

[22] Fanti K (2014). Using a person-entered methodology to investigate the co-occurrence between psychopathological problems. Ann Depress Anxiety.

[23] Wu KK, Cheung MW (2006). Posttraumatic stress after a motor vehicle accident: a six-month follow-up study utilizing latent growth modeling. J Trauma Stress.

[24] Sterling M, Hendrikz J, Kenardy J (2010). Compensation claim lodgement and health outcome developmental trajectories following whiplash injury: a prospective study. Pain..

[25] Galatzer-Levy IR, Huang SH, Bonanno GA (2018). Trajectories of resilience and dysfunction following potential trauma: a review and statistical evaluation. Clin Psychol Rev.

[26] Bryant RA, Nickerson A, Creamer M, O’donnell M, Forbes D, Galatzer-Levy I (2015). Trajectory of post-traumatic stress following traumatic injury: 6-year follow-up. Br J Psychiatry.

[27] Craig A, Tran Y, Guest R, Middleton J (2019). Trajectories of self-efficacy and depressed mood and their relationship in the first 12 months following spinal cord injury. Arch Phys Med Rehabil.

[28] Shakespeare-Finch J, Armstrong D (2010). Trauma type and posttrauma outcomes: differences between survivors of motor vehicle accidents, sexual assault, and bereavement. J Loss Trauma.

[29] O’Donnell ML, Creamer M, Pattison P (2004). Posttraumatic stress disorder and depression following trauma: understanding comorbidity. Am J Psychiatry.

[30] Breslau N, Davis GC, Peterson EL, Schultz LR (2000). A second look at comorbidity in victims of trauma: the posttraumatic stress disorder–major depression connection. Biol Psychiatry.

[31] Schindel-Allon I, Aderka I, Shahar G, Stein M, Gilboa-Schechtman E (2010). Longitudinal associations between post-traumatic distress and depressive symptoms following a traumatic event: a test of three models. Psychol Med.

[32] Heron-Delaney M, Kenardy J, Charlton E, Matsuoka Y (2013). A systematic review of predictors of posttraumatic stress disorder (PTSD) for adult road traffic crash survivors. Injury..

[33] McWilliams LA, Cox BJ, Enns MW (2003). Mood and anxiety disorders associated with chronic pain: an examination in a nationally representative sample. Pain..

[34] Arola HM, Nicholls E, Mallen C, Thomas E (2010). Self-reported pain interference and symptoms of anxiety and depression in community-dwelling older adults: can a temporal relationship be determined?. Eur J Pain.

[35] Vaughan CA, Miles JN, Eisenman DP, Meredith LS (2016). Longitudinal associations among pain, posttraumatic stress disorder symptoms, and stress appraisals. J Trauma Stress.

[36] Duckworth MP, Iezzi T (2010). Physical injuries, pain, and psychological trauma: pathways to disability. Psychol Inj Law.

[37] Olfson M, Gameroff MJ (2007). Generalized anxiety disorder, somatic pain and health care costs. Gen Hosp Psychiatry.

[38] Hooten WM. Chronic Pain and Mental Health Disorders: Shared Neural Mechanisms, Epidemiology, and Treatment. Mayo Clin Proc. 2016;91(7):955–70. 10.1016/j.mayocp.2016.04.029. 10.1016/j.mayocp.2016.04.029 27344405

[39] Means-Christensen AJ, Roy-Byrne PP, Sherbourne CD, Craske MG, Stein MB (2008). Relationships among pain, anxiety, and depression in primary care. Depress Anxiety.

[40] Dueñas M, Ojeda B, Salazar A, Mico JA, Failde I (2016). A review of chronic pain impact on patients, their social environment and the health care system. J Pain Res.

[41] Haythornthwaite J (2006). Assessment of pain beliefs, coping and function. Textbook of pain.

[42] Prastika D, Kitrungrote L, Damkliang J (2016). Pain intensity and pain interference among trauma patients: a literature review. BNJ..

[43] Archer KR, Abraham CM, Obremskey WT (2015). Psychosocial factors predict pain and physical health after lower extremity trauma. Clin Orthop Related Res®.

[44] Platts-Mills TF, Burke GF, Lee YM, Swor RA, Zaleski EZ, Clauw DJ (2012). Pain and interference of pain with function and mood in elderly adults involved in a motor vehicle collision: a pilot study. Exp Aging Res.

[45] Miettinen T, Kautiainen H, Mäntyselkä P, Linton SJ, Kalso E. Pain interference type and level guide the assessment process in chronic pain: Categorizing pain patients entering tertiary pain treatment with the Brief Pain Inventory. PLoS ONE. 2019;14(8):e0221437. 10.1371/journal.pone.0221437. 10.1371/journal.pone.0221437 PMC6701883 31430355

[46] Polinder S, Haagsma J, Bos N, Panneman M, Wolt KK, Brugmans M (2015). Burden of road traffic injuries: disability-adjusted life years in relation to hospitalization and the maximum abbreviated injury scale. Accid Anal Prev.

[47] Harris PA, Taylor R, Thielke R, Payne J, Gonzalez N, Conde JG (2009). Research electronic data capture (REDCap)—a metadata-driven methodology and workflow process for providing translational research informatics support. J Biomed Inform.

[48] Jagnoor J, Blyth F, Gabbe B, Derrett S, Boufous S, Dinh M (2014). Factors influencing social and health outcomes after motor vehicle crash injury: an inception cohort study protocol. BMC Public Health.

[49] EuroQol Group (1990). EuroQol-a new facility for the measurement of health-related quality of life. Health Policy.

[50] Gandek B, Ware JE, Aaronson NK, Apolone G, Bjorner JB, Brazier JE (1998). Cross-validation of item selection and scoring for the SF-12 health survey in nine countries: results from the IQOLA project. J Clin Epidemiol.

[51] Jensen MP, Turner JA, Romano JM, Fisher LD (1999). Comparative reliability and validity of chronic pain intensity measures. Pain..

[52] Sullivan MJ, Bishop SR, Pivik J (1995). The pain catastrophizing scale: development and validation. Psychol Assess.

[53] Creamer M, Bell R, Failla S (2003). Psychometric properties of the impact of event scale—revised. Behav Res Ther.

[54] Beck JG, Grant DM, Read JP, Clapp JD, Coffey SF, Miller LM (2008). The impact of event scale-revised: psychometric properties in a sample of motor vehicle accident survivors. J Anxiety Disord.

[55] Crawford J, Cayley C, Lovibond PF, Wilson PH, Hartley C (2011). Percentile norms and accompanying interval estimates from an Australian general adult population sample for self-report mood scales (BAI, BDI, CRSD, CES-D, DASS, DASS-21, STAI-X, STAI-Y, SRDS, and SRAS). Aust Psychol.

[56] Henry JD, Crawford JR (2005). The short-form version of the depression anxiety stress scales (DASS-21): construct validity and normative data in a large non-clinical sample. Br J Clin Psychol.

[57] Muthén B, Muthén BO (2017). Statistical analysis with latent variables. Mplus User's guide. Eighth edition.

[58] Nylund KL, Asparouhov T, Muthén BO (2007). Deciding on the number of classes in latent class analysis and growth mixture modeling: a Monte Carlo simulation study. Struct Equ Modeling.

[59] Thomas N (2013). Responding to mental health’s mind–body problem. Aust N Z J Psychiatry.

[60] Walker FR, Pfingst K, Carnevali L, Sgoifo A, Nalivaiko E (2017). In the search for integrative biomarker of resilience to psychological stress. Neurosci Biobehav Rev.

[61] Grant GM, O’donnell ML, Spittal MJ, Creamer M, Studdert DM (2014). Relationship between stressfulness of claiming for injury compensation and long-term recovery: a prospective cohort study. JAMA Psychiatry.

[62] Bromet E, Sonnega A, Kessler RC (1998). Risk factors for DSM-III-R posttraumatic stress disorder: findings from the National Comorbidity Survey. Am J Epidemiol.

[63] Flory JD, Yehuda R (2015). Comorbidity between post-traumatic stress disorder and major depressive disorder: alternative explanations and treatment considerations. Dialogues Clin Neurosci.

[64] Craig A, Perry KN, Guest R, Tran Y, Dezarnaulds A, Hales A (2015). Prospective study of the occurrence of psychological disorders and comorbidities after spinal cord injury. Arch Phys Med Rehabil.

