# Supplementary figures and images for: Outcomes after traffic injury: mental health comorbidity and relationship with pain interference
Source: BMC Psychiatry. 2020 Apr 28;20:189. doi: 10.1186/s12888-020-02601-4 (PMC7189452; doi:10.1186/s12888-020-02601-4)

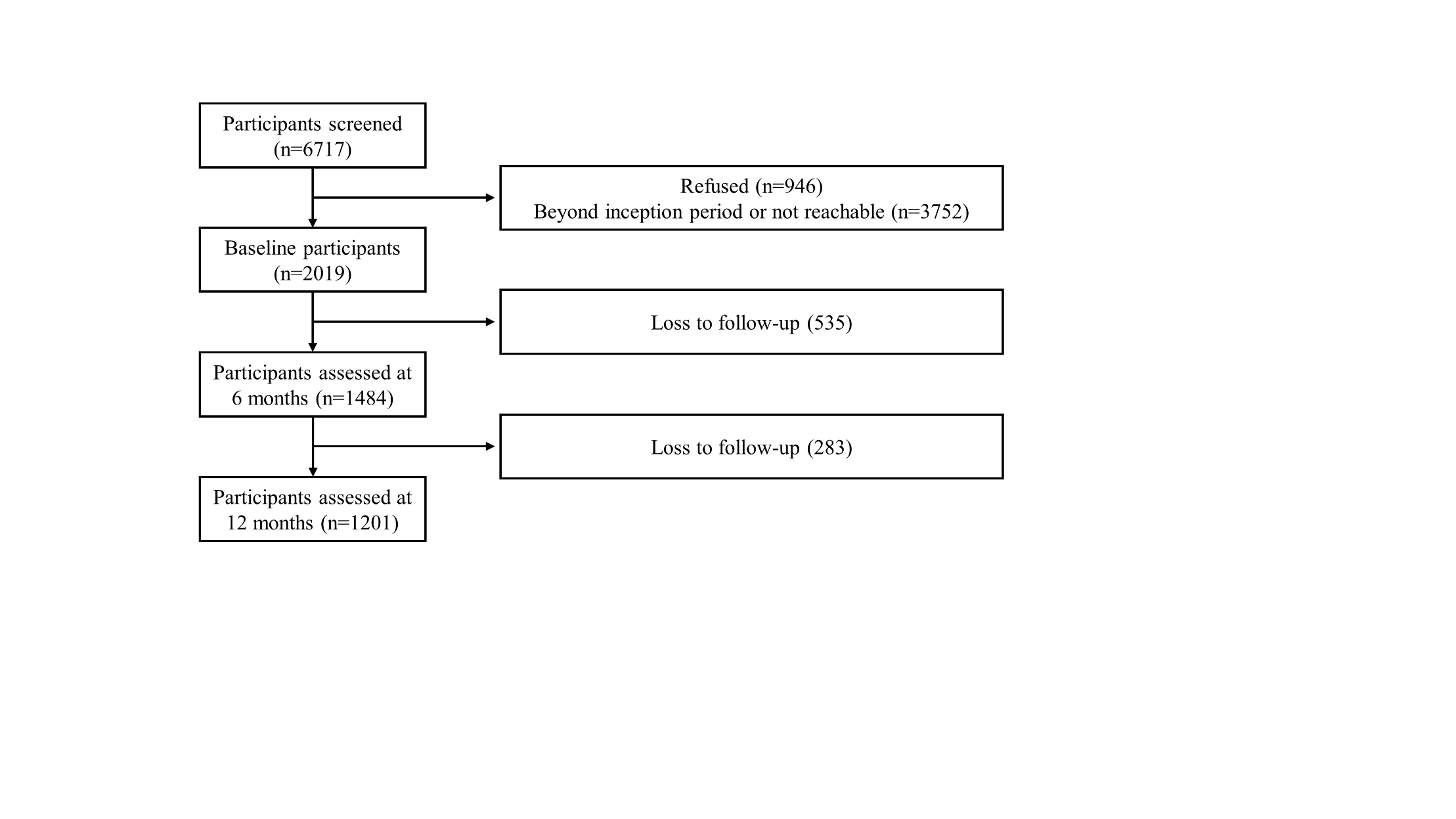
**Additional file 1*.*** Flowchart of study participation.

Supplement: Supplementary file 1 — Additional file 1. Flowchart of study participation. [file 12888_2020_2601_MOESM1_ESM.docx]
