# Supplementary material for: Outcomes after traffic injury: mental health comorbidity and relationship with pain interference
Source: BMC Psychiatry. 2020 Apr 28;20:189. doi: 10.1186/s12888-020-02601-4 (PMC7189452; doi:10.1186/s12888-020-02601-4)
Supplement: Supplementary file 2 — Additional file 2. Fit Indices for 3–6 trajectory growth mixture models for DASS depressive mood (DM), IES-R post-traumatic stress (PTS), and SF-12 pain interference (PI). Five trajectories were chosen as the best fit for DM, four trajectories for PTS, and three for PI. [file 12888_2020_2601_MOESM2_ESM.docx]

**Additional file 2.** Fit Indices for 3-6 trajectory growth mixture models for DASS depressive mood (DM), IES-R post-traumatic stress (PTS), and SF-12 pain interference (PI). Five trajectories were chosen as the best fit for DM, four trajectories for PTS, and three for PI.

|  | Log L | AIC | BIC | Entropy | VLMR LRT p value | LMR adj. LRT p value | PB-LRT  p value |
| --- | --- | --- | --- | --- | --- | --- | --- |
| DM |  |  |  |  |  |  |  |
| 3 trajectories | -11790 | 23626 | 23754 | 0.93 | 0.0008 | 0.0009 | <0.0001 |
| 4 trajectories | -11385 | 22840 | 23036 | 0.91 | 0.14 | 0.14 | <0.0001 |
| 5 trajectories | **-11229** | **22537** | **22755** | **0.91** | **0.017** | **0.018** | **<0.0001** |
| 6 trajectories | -11055 | 22211 | 22490 | 0.91 | 0.12 | 0.13 | <0.0001 |
| PTS |  |  |  |  |  |  |  |
| 3 trajectories | -8970 | 17990 | 18130 | 0.85 | 0.003 | 0.003 | <0.0001 |
| 4 trajectories | **-8958** | **17974** | **18136** | **0.86** | **0.018** | **0.019** | **<0.0001** |
| 5 trajectories | -8846 | 17754 | 17927 | 0.83 | 0.13 | 0.13 | <0.0001 |
| 6 trajectories | -8727 | 17534 | 17757 | 0.83 | 0.2 | 0.2 | <0.0001 |
| PI |  |  |  |  |  |  |  |
| 2 trajectories | -2534 | 5079 | 5108 | 0.64 | <0.0001 | <0.0001 | <0.0001 |
| 3 trajectories | **-2529** | **5065** | **5082** | **0.76** | **<0.0001** | **<0.0001** | **<0.0001** |
| 4 trajectories | -2529 | 5067 | 5090 | 0.67 | <0.0001 | <0.0001 | <0.0001 |
| 5 trajectories | -2529 | 5069 | 5097 | 0.45 | 0.001 | 0.003 | <0.0001 |

Note: adj: adjusted; LRT: Likelihood ratio test; VLMR (Vuong-Lo-Mendell-Rubin LRT); MR (Lo-Mendell-Rubin adjusted LRT test); PBLRT (Parametric bootstrapped likelihood ratio test).
