# Supplementary material for: Outcomes after traffic injury: mental health comorbidity and relationship with pain interference
Source: BMC Psychiatry. 2020 Apr 28;20:189. doi: 10.1186/s12888-020-02601-4 (PMC7189452; doi:10.1186/s12888-020-02601-4)
Supplement: Supplementary file 3 — Additional file 3. Predictors of depressive mood (DM) and post-traumatic stress (PTS) trajectories. [file 12888_2020_2601_MOESM3_ESM.docx]

**Additional file 3.** Significant predictors of depressive mood (DM) and post-traumatic stress (PTS) trajectories.

| Predictors of DM trajectories | | | | | |
| --- | --- | --- | --- | --- | --- |
|  | Chronic  (Trajectory 1) | Moderate-Chronic (Trajectory 2) | Worsening  (Trajectory 3) | Recovery  (Trajectory 4) | Resilient  (Trajectory 5) |
|  | *Odds ratio (pval)* | *Odds ratio (pval)* | *Odds ratio (pval)* | *Odds ratio (pval)* | *Odds ratio (pval)* |
| Pre-injury health | 0.001*** | 0.002*** | 0.007*** | 0.017*** | 1.0 (ref) |
| Baseline PTS | 2.99*** | 1.82*** | 1.32*** | 1.54*** | 1.0 (ref) |
| Pain catastrophising | 1.08*** | 1.07*** | 1.02* | 1.03** | 1.0 (ref) |
| Social life dissatisfaction | 2.67*** | 2.58*** | 1.34* | 1.62** | 1.0 (ref) |
| Physical health quality of life | 0.98 | 0.98 | 0.94*** | 1.00 | 1.0 (ref) |
| Mental health quality of life | 0.86*** | 0.89*** | 0.95*** | 0.91*** | 1.0 (ref) |
| Predictors of PTS trajectories | | | | | |
|  | Chronic (Trajectory 1) | Recovery (Trajectory 2) | Worsening (Trajectory 3) | Resilient (Trajectory 4) | - |
|  | *Odds ratio (pval)* | *Odds ratio (pval)* | *Odds ratio (pval)* | *Odds ratio (pval)* | - |
| Baseline DM | 1.29*** | 1.17*** | 1.12*** | 1.0 (ref) | - |
| Pain intensity | 1.20* | 1.02 | 1.22** | 1.0 (ref) | - |
| Pain catastrophising | 1.12*** | 1.08*** | 1.05*** | 1.0 (ref) | - |
| Mental health quality of life | 0.94** | 0.96*** | 0.96** | 1.0 (ref) | - |

*p<0.05 **p<0.01 ***p<0.001
