# Supplementary material for: Outcomes after traffic injury: mental health comorbidity and relationship with pain interference
Source: BMC Psychiatry. 2020 Apr 28;20:189. doi: 10.1186/s12888-020-02601-4 (PMC7189452; doi:10.1186/s12888-020-02601-4)
Supplement: Supplementary file 4 — Additional file 4. Joint and conditional probabilities* between post-traumatic stress (PTS) trajectories and pain interference (PI) trajectories. [file 12888_2020_2601_MOESM4_ESM.docx]

**Additional file 4.** Joint and conditional probabilities* between post-traumatic stress (PTS) trajectories and pain interference (PI) trajectories.

|  | PTS trajectories | | | |
| --- | --- | --- | --- | --- |
|  | Chronic  (Trajectory 1) | Worsening  (Trajectory 3) | Recovery  (Trajectory 2) | Resilient  (Trajectory 4) |
| Joint probability of PTS & PI | | | | |
| PI trajectories |  |  |  |  |
| Chronic (Trajectory 1) | 11.1 | 6.7 | 13.3 | 27.6 |
| Recovery (Trajectory 2) | 0.4 | 0.9 | 4.7 | 20.3 |
| Resilient (Trajectory 3) | 0.4 | 0.2 | 1.4 | 13.0 |
|  |  |  |  |  |
| Conditional probability of PTS given PI/ of PI given PTS | | | | |
| PI trajectories |  |  |  |  |
| Chronic (Trajectory 1) | 18.9/ **93.2** | 11.4/ **85.7** | 22.7/ **68.7** | 47.0/ **45.3** |
| Recovery (Trajectory 2) | 1.5/ **3.4** | 3.3/ **11.0** | 17.9/ **24.2** | 77.3/ **33.3** |
| Resilient (Trajectory 3) | 2.7/ **3.4** | 1.7/ **3.2** | 9.1/ **7.0** | 86.6/ **21.4** |

* Probabilities are expressed in %

Note: Figures in bold pertain to conditional probability of PI given PTS.
